# The plasticity of epithelial cells and its potential in the induced differentiation of gastric cancer

**DOI:** 10.1038/s41420-024-02275-x

**Published:** 2024-12-24

**Authors:** Ziwei Yan, Yingnan Liu, Yuan Yuan

**Affiliations:** 1 Tumor Etiology and Screening Department of Cancer Institute and General Surgery, The First Hospital of China Medical University, Shenyang, Liaoning People’s Republic of China; 2 Key Laboratory of Cancer Etiology and Prevention in Liaoning Education Department, The First Hospital of China Medical University, Shenyang, China; 3 Key Laboratory of GI Cancer Etiology and Prevention in Liaoning Province, The First Hospital of China Medical University, Shenyang, China

**Keywords:** Cancer prevention, Mechanisms of disease, Targeted therapies

## Abstract

Cell plasticity refers to the deviation of cells from normal terminal differentiation states when faced with environmental and genetic toxic stresses, resulting in the phenomenon of transforming into other cell or tissue phenotypes. Unlocking phenotype plasticity has been defined as a hallmark of malignant tumors. The stomach is one of the organs in the body with the highest degree of self-renewal and exhibits significant cell plasticity. In this paper, based on the review of the characteristics of normal differentiation of gastric epithelial cells and their markers, the four main phenotypes of gastric epithelial cell remodeling and their relationship with gastric cancer (GC) are drawn. Furthermore, we summarize the regulatory factors and mechanisms that affect gastric epithelial cell plasticity and outline the current status of research and future prospection for the treatment targeting gastric epithelial cell plasticity. This study has important theoretical reference value for the in-depth exploration of epithelial cell plasticity and the tumor heterogeneity caused by it, as well as for the precise treatment of GC.

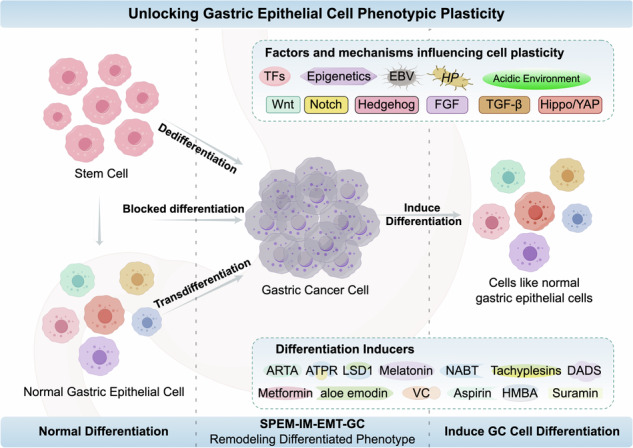

## Facts

- Cellular plasticity refers to the determination, transformation, and reshaping of cell fate. Tumor cells’ plasticity allows them to evade immune attacks and resist anti-tumor treatments, enhancing their survival.
- The stomach exhibits significant cell plasticity, with differentiated cells able to reprogram into other mature cell types, this plasticity can lead to cycles of differentiation and dedifferentiation, increasing the risk of cancer-prone mutations.
- The phenotype of gastric epithelial cells is reshaped through transdifferentiation, dedifferentiation, and blocked differentiation, showing different differentiation phenotypic characteristics.
- The activation of transcription factors, abnormal epigenetic regulation, infection, acidic environment, and the combined effect of signaling pathways such as Wnt, Notch, Hedgehog, etc., constitute a complex network that directly or indirectly regulates the plasticity of gastric epithelial cells.
- GC treatment strategies based on the mechanism of cellular plasticity have become a research hot spot, including the development of small molecules, organic compounds, hormones, etc.

## Open questions

- How can we establish more accurate and effective in vivo and in vitro models to simulate the dynamic changes and spatiotemporal distribution of gastric epithelial cell plasticity?
- How can we utilize single-cell and multi-omics analyses to distinguish individual differences and subtypes within the plasticity of gastric epithelial cells?
- How can we harness the plasticity of gastric epithelial cells to develop novel prevention, diagnosis, and treatment strategies for GC?
- How can we assess and apply the plasticity of gastric epithelial cells in clinical treatment for GC patients to improve therapeutic outcomes?

## Introduction

Cell differentiation is the cytological basis of individual development. During development, cells of the same origin gradually differentiate into different levels, giving rise to cell populations with different morphological structures and functional characteristics, thus constructing the tissues and organs necessary for multicellular life [1, 2]. The phenomenon of cells deviating from normal terminal differentiation states to acquire other cell or tissue phenotypes in response to environmental and genetic toxic stress is known as cell plasticity. Cell plasticity is an important fundamental characteristic of cell development and tissue regeneration, and unlocking phenotype plasticity has been defined as the latest conceptual hallmark of malignant tumors [3]. Moreover, tumor cells’ plasticity allows them to resist immune attacks as well as anti-tumor treatments, which promotes their survival.

The stomach, as one of the organs with the highest degree of self-renewal in the body, exhibits significant cell plasticity. Differentiated mature cells can be reprogrammed into another mature cell as a reserve stem cell to replace lost cells. This plasticity implies that gastric epithelial cells may undergo cycles of differentiation and dedifferentiation, thereby increasing the risk of accumulating cancer-prone mutations [4]. Gastric cancer (GC) is a malignant tumor originating from the gastric mucosal epithelium and is the fifth most common malignancy globally [5]. The occurrence of GC is closely related to the differentiation of gastric epithelial cells, and the degree of differentiation of GC cells has been shown to be an important factor in predicting the survival of GC patients [6].

Previous research reviews have shed light on specific aspects of gastric cell biology, such as the roles of gastric stem cells (Meng Liu et al. [7]), the process of metaplasia (James R Goldenring et al. [8]), and the mechanisms of epithelial-mesenchymal transition (EMT) (Dandan Li et al. [9]), which have provided us with valuable information, but they have also left some gaps because they focus on the detailed mechanisms of individual processes involved in phenotypic transformation of gastric epithelial cells from a relatively single perspective. The uniqueness of this review lies in the fact that we not only review the characteristics and markers of normal differentiation of gastric epithelial cells but also, based on the concept of unlocking phenotypic plasticity proposed in the latest edition of the Hallmarks of Cancer: New Dimensions [3], we focus on the four main phenotypes of gastric epithelial cell remodeling and their relationship with the occurrence of GC. Furthermore, we have further summarized the regulatory factors and mechanisms affecting the plasticity of gastric epithelial cells, and combed through the treatment strategies, current research status, and future research directions related to the plasticity of gastric epithelial cells, and proposed the challenges and issues faced in this field. These contents may not have been discussed so systematically in previous reviews, and we believe they can provide important theoretical reference value for a deeper understanding of the plasticity of gastric epithelial cells, the tumor heterogeneity it causes, and the precision treatment of GC. This study has important theoretical reference value for exploring the tumor heterogeneity caused by epithelial cell plasticity and for the precise diagnosis/treatment of GC, providing important scientific basis for a more comprehensive understanding and intervention of the occurrence and development of GC.

## Normal differentiation characteristics and markers of gastric epithelial cells

### Normal differentiation process of gastric epithelial cells

The normal development and differentiation of gastric epithelial cells is a complex and dynamic biological process involving multiple stages of differentiation and regulatory mechanisms. Initially, precursors of gastric epithelial cells originate from the foregut region of the endoderm, where multipotent progenitor cells are regulated by core transcription factors such as FoxA, Gata, Sox17, and Mixl1, which play crucial roles in the proliferation and survival of foregut progenitor cells [10]. As the endoderm gradually specializes, cellular lineages become more defined, with foregut progenitor cells further differentiating into progenitors of the anterior stomach and gastric body. Pdx1 expression marks anterior stomach progenitors as SOX2 + GATA4 + PDX1+ and gastric body progenitors as SOX2 + GATA4 + PDX1-. The absence of Pdx1 leads to anomalies in anterior stomach progenitors, resulting in abnormalities in the pyloric structure and loss of gastrin-endocrine cells [7]. During endoderm specialization, as developmental signals change, the gastric epithelium further differentiates and forms primitive gastric gland structures. These small glands invaginate from the single-layer epithelium into the mesenchyme, ultimately forming the basic gastric gland units, including isthmus cells, chief cells, and mucosal surface cells [11]. Different types of cells have specific distribution and function within the gastric glands, working together to perform the stomach’s digestive and protective functions.

In adulthood, gastric epithelial cells maintain homeostasis and tissue integrity through dynamic self-renewal mechanisms. Multipotent stem cells are located in the basal region of the gastric glands, with Lgr5+ cells being considered an important subgroup of stem cells that can undergo symmetric division and maintain the self-renewal state of stem cells. The SOX2+ and LGR5+ cell populations together constitute the gastric gland stem cell reservoir, which can continuously differentiate into various types of gastric epithelial cells to replace aged or damaged cells [12]. Lineage tracing studies have shown that SOX2+ cells have the long-term ability to generate different types of gastric epithelial cells [13]. Additionally, the renewal of gastric epithelial cells is not only dependent on stem cells but can also be supplemented through dedifferentiation of terminally differentiated mature cells, thereby enhancing tissue repair capabilities.

Through these precise regulatory mechanisms, the homeostasis of gastric epithelial cells is maintained to ensure the normal function of the stomach and rapid repair of damage. The complexity and fine-tuning of this process make it crucial for studying the mechanisms of gastric diseases and GC development [14, 15].

### Markers of differentiation and maturation of gastric mucosal epithelial cells

The molecular markers of gastric epithelial cell maturation refer to genes or proteins that can reflect the differentiation state and type as well as specific physiological roles of gastric epithelial cells. Their presence and activity are usually associated with cells that have completed the differentiation process, mature status, and normal maintenance of gastric function. Different types of cells maintain the normal physiological status of the entire stomach through their unique functions. Currently, several major molecular and functional markers of gastric epithelial cell maturation have been discovered, including Mist1, PGA, PGC, Gastrin, MUC5AC, MUC6, MUC17, SOX2, TFF2, Hydrochloric Acid, Intrinsic Factor, H+、K+-ATPase etc. (Table 1). Those markers are vital for studying the stomach’s development, homeostasis, and diseases, providing insights into the biological characteristics of gastric epithelial cells.

**Table 1:** Molecular and functional markers of gastric epithelial cell differentiation and maturation.

Marker	Function	Resourse	Regulatory Signals	Reference
Mist1	bHLH transcription factor, plays a crucial role in the differentiation and function of gastric chief cells	chief cells	Gastric microenvironment and signaling molecules.	[46, 166]
PGA	Directly participates in the digestion process of proteins, and serves as an indicator of gastric mucosal status	mucous neck cells of the chief cells	Gastric acid secretion, the state of the gastric mucosa, and external irritation	[167]
PGC	PGC levels represent the quantity of gastric mucosal glands and cells; reduced levels indicate the degree of gastric mucosal atrophy	chief cells	Gastric acid concentration, gastric mucosal status, and external factors	[167]
Gastrin	Peptide hormone secreted by gastric antral G cells, functional marker for the gastric antrum	G cells	Stimulated by food, particularly intake of proteins and peptides	[168, 169]
MUC5AC	Participate in processes such as cell growth and differentiation, intercellular signal transduction, and intercellular adhesion, playing a protective and lubricating role for the mucosal epithelium	Surface pits and mucous neck cells	Pathological conditions of the stomach, hormonal and neuronal factors, as well as environmental factors	[170]
MUC6	Mucin protein secreted by neck mucous cells in the gastric fundus and body	neck mucous cells	Pathological conditions of the stomach, hormonal and neuronal factors, as well as environmental factors	[170, 171]
MUC17	Mucin protein secreted by surface mucous cells in the gastric antrum	neck mucous cells	Pathological conditions of the stomach, hormonal and neuronal factors, as well as environmental factors	[172]
SOX2	Plays a key role in the maintenance and differentiation of gastric epithelial cells	gastric epithelial stem cells and progenitor cells	Gastric microenvironment and signaling molecules.	[173, 174]
TFF2	Peptide molecule belonging to the TFF family, secreted by granule cells in the gastric antrum	granule cells	Gastric microenvironment and signaling molecules.	[175]
Hydrochloric Acid	Activates pepsinogen, promotes the generation of pepsin, has bactericidal properties, and stimulates secretion	parietal cells	Signals from histamine, acetylcholine, and gastrin	[176, 177]
Intrinsic Factor	Binds with vitamin B12, facilitates absorption in the ileum, and supports red blood cell production	parietal cells	Signals from histamine, acetylcholine, and gastrin	[178]
H+、K+-ATPase	Proton discharge into the stomach, combining with chloride ions to form hydrochloric acid, facilitates gastric acid generation	parietal cells	Nerves, hormones, and food intake	[179]

## Remodeling phenotype of gastric epithelial cells and its relationship with GC

During the development and maturation of gastric mucosal tissue, terminal differentiation can be interrupted due to the influence of various factors. Gastric epithelial cell proliferation potential and phenotype plasticity are reactivated simultaneously. Through transdifferentiation (cells crossing differentiation pathways from one mature state to another), dedifferentiation (lineage reversal), and blocked differentiation [16], the phenotype of gastric epithelial cells is reshaped.

### Transdifferentiation phenotypes

#### Spasmolytic polypeptide-expressing metaplasia

Spasmolytic polypeptide-expressing metaplasia (SPEM) is a mechanism that responds to glandular damage and is evolutionarily conserved, which is commonly found in the gastric fundus. Its essence is that epithelial cells are transformed into cells with the characteristics of gastric gland cells, which allows cells to adapt to damage from gastric acid [17]. SPEM represents a metaplastic reaction unique to the gastric corpus, given the absence of parietal and chief cells in the gastric antrum [18]. A loss of parietal cells, additional injury, and/or specific changes in immune and stromal cells along the gland axis may be required to initiate SPEM. Various epithelial factors (e.g., NOTCH ½ [19], Shh [20]), stromal factors (e.g., BMP-4 [21], noggin [22]), and immune factors (e.g., IFNγ [23], IL-1β [24]) contribute to the establishment of the SPEM metaplastic environment and may be involved in the loss of parietal cells or induction of SPEM. SPEM can cause intestinal metaplasia (IM) and is even more strongly associated with GC than IM [25]. Clinical studies have shown that SPEM can be found in 90% of GCs resected by surgery [26]. Therefore, the relationship between SPEM and GC is quite close.

#### Intestinal metaplasia

IM is essentially a form of transdifferentiation involving the transformation of gastric mucosal epithelial cells into intestinal-type epithelial cells. IM can be categorized as complete IM and incomplete IM. Complete IM resembles small intestinal gland, being free of gastric mucin (MUC1, MUC5AC, and MUC6) and the presence of acidophilic intestinal cells, accompanied by brush borders, well-defined goblet cells, and occasional Paneth cells. Contrary to this, the structure of incomplete IM (also called gastric-type or mixed-type IM) resembles that of colonic glands, with numerous irregular cytoplasmic mucin droplets and a lack of brush borders. It is also often found to express gastric and intestinal mucin simultaneously [27]. Incomplete IM is a more important risk factor for GC compared to complete IM [28]. The molecular characteristics of IM in the stomach include the ectopic expression of the CDX family of transcription factors CDX1 and CDX2. Complete IM exhibits the highest CDX2 protein expression, followed by an incomplete IM, with the lowest expression in GC [29, 30]. Meta-analysis shows that IM increases the risk of GC by 3.6 times [31]. The median time for the progression of IM to GC is 6.1 years [32], providing an opportunity for the identification and preventive intervention of high-risk individuals during the precancerous latent period.

### Transdifferentiation + dedifferentiation phenotypes

#### Epithelial-mesenchymal transition

The epithelial-mesenchymal transition (EMT), in essence, is a biological process in which epithelial cells undergo transformation into mesenchymal cells [33]. Rather than a simple shift from epithelial to mesenchymal or fibroblast cells, EMT involves a complex mix of transdifferentiation and dedifferentiation processes [34]. EMT is viewed as a crucial process during which cancer cells originating from epithelial tissue gain the capacity to migrate and invade surrounding tissues [35, 36]. At the same time, migrating and invasive mesenchymal-like cells often acquire cancer stem cell (CSC) characteristics and are resistant to treatment, resulting in a poor prognosis [37]. Additionally, to confer the ability to metastasize directly to tumor cells, EMT also promotes tumor metastasis indirectly by enhancing stemness, immune evasion, and chemotherapy resistance [38]. These research findings indicate that EMT signals play a key role in directly and indirectly promoting GC progression.

### Dedifferentiation + blocked differentiation phenotype

#### GC

The existence of cancer stem cells (CSCs) is a major cause of GC occurrence and heterogeneity. CSCs are a highly plastic state that allows tumor cells to rapidly adapt to various conditions in the tumor microenvironment [39]. The dedifferentiation of short-lived progenitor cells or mature cells into long-lived stem cells can result in a cancerous state [40, 41]. Gastric stem cells with Apc mutations produce mucosal dysplasia or adenomas in the distal stomach [42, 43], and a combination of carcinogenic drivers such as Apc, p53, Kras, or Smad induces invasive cancer originating from antral stem cells [44, 45]. Thus, it is necessary and sufficient for the progressive development of GC for gene mutations to induct and accumulate in gastric antrum stem cells. The cell origins of gastric tumors are both basal and +4 gastric antrum stem cells in models using Helicobacter pylori (Hp) infection and MNU. Specific induction of carcinogenic mutations in isthmus stem cells has also been shown to contribute to GC formation. Upon chronic inflammation induced by Hp infection, Mist1-derived clones that lack Cdh1 survive and expand, ultimately giving rise to GCs that resemble human signet rings [46]. Given that Hp infection and Kras mutations in the stomach both lead to SPEM and IM, these pathological changes may be necessary for the development of GC originating from isthmus stem cells [47].

## Factors and mechanisms affecting gastric epithelial cell plasticity

### Factors affecting gastric epithelial cell plasticity

#### Transcriptional factor regulation

Transcription factors, a class of proteins that bind to DNA and regulate gene expression, are vital for cell differentiation, development, and functional regulation. For gastric epithelial cells, transcription factors are essential for maintaining cell phenotype and function.

When gastric epithelial cells undergo plastic changes, specific transcription factors, such as members of the GATA, RUNX, and CDX families, may be activated or suppressed, thus guiding cells in a particular direction of development (Table 2).

**Table 2:** Transcription factors affecting gastric epithelial cell plasticity.

Transcription factor	Specificity/role in GC	Impact on cell plasticity	Mechanism	Reference
GATA2	Heterogeneous expression in GC.	Loss of expression creates a favorable environment for GC growth and survival. Regulates GC cell chemotherapy resistance.	Abnormal epigenetic dysregulation of GATA2 induces GATA2/GATA6 conversion in GC and promotes GATA6 expression to promote GC. CGA/EGFR/GATA2 forms a positive feedback loop to induce chemotherapy resistance.	[180–182]
GATA3	Directs normal pyloric development and is low expressed in GC cells.	Regulates GC cell growth and stemness.	Affects the expression of Connexin43 and Connexin 32 and regulates the occurrence of Hp-related GC. USP21 combines with GATA3 to regulate the expression of MAPK1.	[183–188]
GATA4	Essential for the formation of gastric mucosa and maintaining its normal tissue renewal and differentiation. Abnormally highly expressed in GC cells.	Regulates GC cell proliferation and self-renewal.	KLF5/GATA4/GATA6 crosstalk promotes GC development and collaboratively maintains the cancer-promoting transcriptional regulatory network. Forming a positive feedback loop with CDX2 to transactivate MUC2 in intestinal metaplasia. Methylation occurs in the early stages of chronic gastritis and GC, and its methylation affects GC cell proliferation and apoptosis.	[189–193]
GATA6	Essential for the formation of gastric mucosa and maintaining its normal tissue renewal and differentiation. Abnormally highly expressed in GC cells.	Promote the occurrence of EMT in GC cells. Promotes GC cell proliferation. Promotes stem cell properties in GC cells. Regulates drug resistance in GC cells.	Acts synergistically with other transcription factors. Regulates gene expression related to cell proliferation, differentiation, and cancer progression. ΔNp63α upregulates GATA6 to mediate human GC cell proliferation and apoptosis. Regulates expression of mucin Muc5ac.	[180, 193–196]
RUNX1	Tumor suppressor, low expression in GC.	Down-regulation promotes GC cell proliferation and invasion.	H19/miR-675 axis regulates RUNX1 expression and regulates Akt/mTOR pathway.	[63, 64, 197]
RUNX2	Carcinogenic factor, expressed in GC.	Maintain the self-renewal potential of GC cells. Promotes invasion and transfer potential.	Positive regulation of the YAP signaling pathway.	[198–200]
RUNX3	Controlling the growth and differentiation of gastric epithelial cells helps maintain normal gastric mucosal structure. Tumor suppressor, low expression in GC.	Protects gastric epithelial cells from aberrant growth factor signaling and resulting cellular plasticity and stemness. Modulates GC cell drug resistance.	Acts as both a mediator of TGF-β signaling and an antagonist of Wnt. Downregulating the expression of apoptosis-related genes Bcl-2, MDR-1 and MRP-1 makes GC cells sensitive to chemotherapy drugs.	[201–208]
CDX1	Absent in normal gastric mucosa, associated with intestinal phenotype.	Induce the transformation of gastric epithelial cells into tissue stem cell-like progenitor cells, followed by transdifferentiation into intestinal epithelial cells.	Induces abnormal expression of stemness-related reprogramming factors SALL4 and KLF5.	[30, 209–212]
CDX2	Absent in normal gastric mucosa, associated with intestinal phenotype.	Abnormally expressed and plays a key role in intestinal metaplasia of gastric epithelium.	Rapidly induced intestinal differentiation.	[30, 173, 209, 211–215]

#### Epigenetic regulation

Epigenetic factors play an important role in regulating gastric epithelial cell plasticity, responding to physiological stimuli and disease, and undergoing cell fate transitions and reprogramming events during development [48]. Epigenetic factors can also act as intermediaries between external stimuli and gene expression regulation [49]. Epigenetic regulation mainly includes DNA methylation and histone modifications (such as acetylation, methylation, etc.), as well as non-coding RNAs in various forms [50, 51]. These mechanisms not only independently affect cell behavior but also interact with each other to form a coordinated network that collectively influences the plasticity of gastric epithelial cells.

DNA methylation, as one of the core mechanisms of epigenetic regulation, is essential in embryonic development, tissue formation, and the maintenance of stable cell lines and phenotypes [52]. In gastric epithelial cells, changes in the methylation patterns of specific genes may be closely related to their differentiation, proliferation, and regenerative capabilities. Kristin Fritsche et al. found that differences in DNA methylation between different gastric regions could be transformed into regulatory effects on the transcriptome phenotype [53]. Abnormal DNA methylation is a hallmark of glandular epithelium transformation causing gastric adenocarcinoma [54], and changes in DNA methylation can be observed in precancerous lesions, including IM [55]. These findings suggest that changes in DNA methylation in gastric epithelial cells can affect not only the function of individual genes but also lead to broader changes in cell fate.

Histone modifications, another significant class of epigenetic regulatory mechanisms, can influence gene expression in gastric epithelial cells by altering the structure and function of chromatin, primarily through acetylation, methylation, and phosphorylation. Disruptions in histone modifications can alter the plasticity of GC cells and affect their proliferation, migration, and invasion capabilities [56, 57]. Histone acetylation is a covalent reaction catalyzed by histone acetyltransferases (HATs). Imbalances between HATs and histone deacetylases (HDACs) can cause changes in gastric epithelial cell plasticity [58]. For example, HDAC3 is a subtype of HDACs, and in epithelial cells, hypoxia can induce the expression of HDAC3 and its enzyme activity to remove acetyl groups from lysine 4 of histone H3 (H3K4Ac), promoting the suppression of epithelial genes, activation of mesenchymal gene expression, and inhibition of chromatin structure associated with epithelial genes [59, 60]. Additionally, histone modifications rapidly change in response to cellular damage, mobilizing gene expression related to repair and regeneration.

Non-coding RNAs, as an emerging field in epigenetic regulation, also play an increasingly important role in the modulation of gastric epithelial cell plasticity. NcRNAs and their synergistic interactions with proteins, DNA, and other RNA molecules play a significant role in regulating cell function and plasticity. For example, H19 is a highly conserved lncRNA involved in embryonic development and tissue differentiation [61]. Studies have found that H19 is highly expressed in GC and is associated with the degree of GC differentiation, lymph node metastasis, and prognosis [62]. H19 can regulate the proliferation, migration, and invasion of GC cells by interacting with miR-675 [63, 64]. H19 can also promote the EMT of GC cells and increase the plasticity and stemness of GC cells by interacting with miR-141 [65, 66]. The miR-200 family is downregulated in GC and is associated with the degree of GC differentiation, lymph node metastasis, and prognosis [67]. The miR-200 family can inhibit the EMT of GC cells by targeting transcription factors such as ZEB1 and ZEB2, thereby reducing the plasticity and stemness of GC cells [68, 69]. miR-21 can inhibit the proliferation and invasion ability of GC cells [70], and miR-21, miR-10b, and miR-146a are upregulated in GC stem-like cells [71]. In summary, non-coding RNAs, as one of the important forms of epigenetic regulation, may contribute to the plasticity of gastric epithelial cells through multiple mechanisms. NcRNAs play a significant role in the regulation of GC and the plasticity of gastric epithelial cells, forming a complex epigenetic regulatory network alongside DNA methylation and histone modifications.

#### Infectious factor regulation

In the complex landscape in which GC occurs, understanding the interactions between infectious agents and cellular processes is critical. At present, Helicobacter pylori (HP) and Epstein–Barr virus (EBV) are known to be closely related to GC. These pathogens can, through their unique mechanisms, not only trigger cell transformation, but also interact with the host’s genetic and epigenetic mechanisms, leading to a chain of events that affect cellular plasticity and ultimately lead to the development of precancerous lesions and potential GC.

HP has been classified as a class I carcinogen [72], which can reach the gastric epithelium through complex motility and chemotaxis systems [73]. Its interaction with epithelial cells can lead to changes in cell signaling, DNA damage, and epithelial immune abnormalities [74]. HP colonization near stem cell niches triggers specific inflammation, promoting epithelial pathology. This enhances cell turnover and plasticity, fostering the formation of basal antimicrobial cells [75]. Studies suggest that prolonged exposure of gastric epithelial cells to components secreted by fibroblasts activated by HP can induce precancerous phenotypic reprogramming of the gastric epithelium. This process may be TGFβ-dependent and lead to the reprogramming of gastric epithelial cells towards cancer stem cell-related differentiation programs [76]. The phenotypic plasticity of gastric epithelial cells caused by Hp infection determines their susceptibility to pro-invasive signaling, leading to the reprogramming of the gastric niche and providing clues for the promotion/progression of GC [77]. Furthermore, Hp often interacts with other factors to affect the plasticity of gastric epithelial cells, including the aforementioned transcription factors and epigenetic factors.

EBV can infect B lymphocytes and epithelial cells, and is associated with the development of various tumors, including GC [78, 79]. The direct relationship between EBV infection and the plasticity of gastric epithelial cells has not been widely explored. However, some studies suggest that EBV may influence cell growth and proliferation by interacting with host cells and altering specific cellular signaling pathways. For example, EBV may regulate the immortalization and transformation, proliferation, invasion, apoptosis, and other phenotypes of gastric epithelial cells through its encoded proteins such as LMP1, BARF1, etc., and contribute to the pathogenesis of GC [80, 81]. In addition, EBV may also affect the phenotype of gastric epithelial cells through interactions with miRNAs such as the miR-200 family [82, 83].

#### Acidic environment regulation

The gastric mucosa can regulate the behavior of epithelial cells through acid production to adapt to various forms of endogenous and exogenous damage. From this perspective, a stomach’s damage response can be divided into two main patterns: surface response, which involves the migration and rapid proliferation of surface epithelial cells to repair erosion caused by acid or other irritants; and glandular response, which involves the reprogramming of mature cells to function as auxiliary stem cells to replace lost cells [84]. Additionally, it has been found that when primary cultured gastric cells are briefly exposed to strong acid, body-like cells appear to exhibit plastic characteristics of selectively expressing pluripotent stem cell marker proteins or expressing pluripotent stem cell marker proteins in cell subsets [85].

### Mechanisms affecting gastric epithelial cell plasticity

**Fig. 1:**
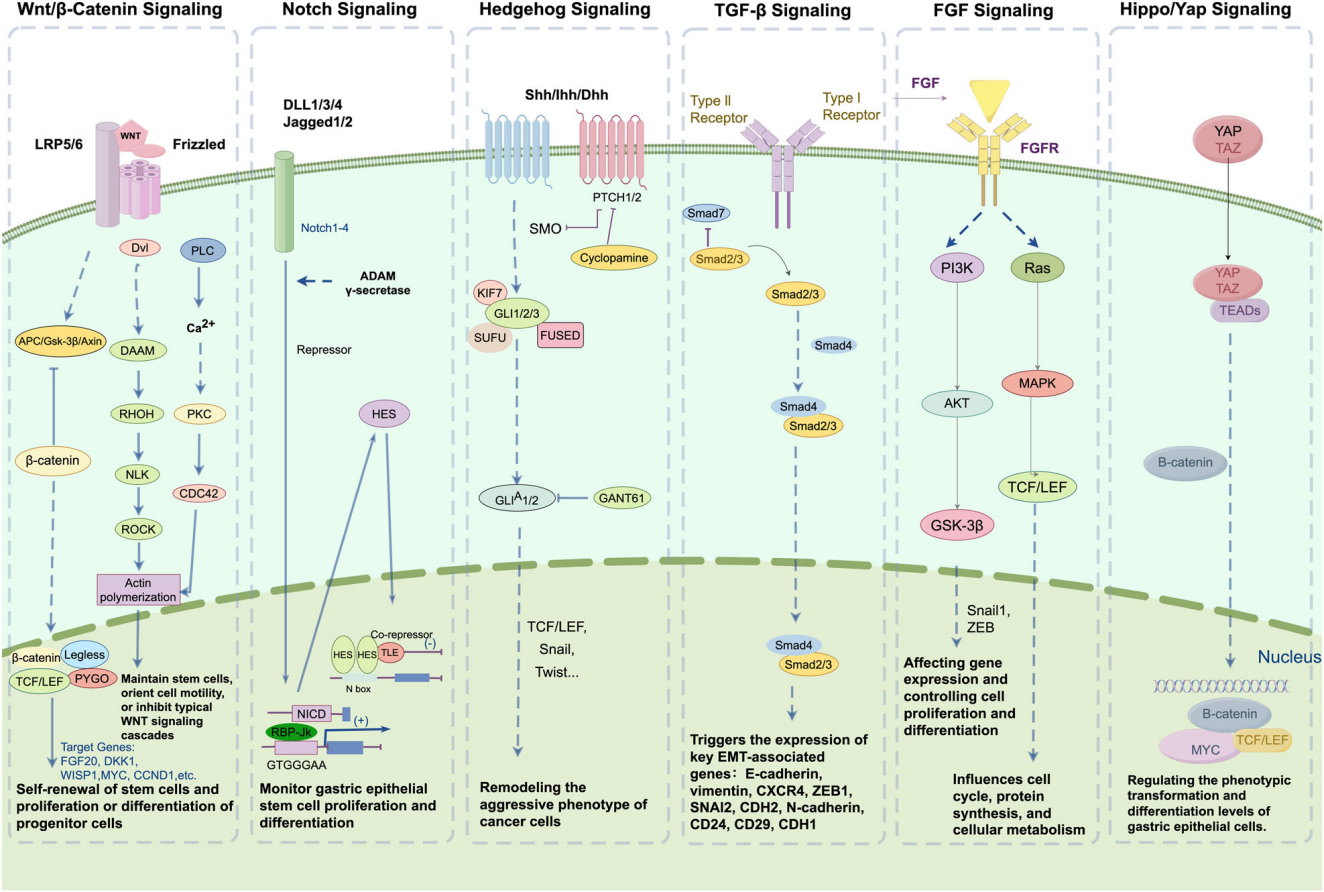
Mechanisms affecting gastric epithelial cell plasticity. Signaling pathways involved in gastric epithelial cell phenotypic transformation. The most important signaling pathways involved in gastric epithelial cell phenotypic transformation include Wnt signaling pathway, Notch signaling pathway, and Hedgehog (Hh) signaling pathway, transforming growth factor-β (TGF-β) signaling pathway, fibroblast growth factor (FGF) signaling pathway and Hippo/YAP signaling pathway. The figure shows pathways that are activated in these processes (by Figdraw).

Multiple signaling pathways play a crucial role in regulating gastric epithelial cell plasticity. Through interactions and cross-regulation with various influencing factors, they collectively form a complex regulatory network governing gastric epithelial cell plasticity. The most important signaling pathways involved in gastric epithelial cell phenotypic transformation include Wnt Signaling Pathway, Notch Signaling Pathway, and Hedgehog (Hh) signaling pathway, transforming growth factor-β (TGF-β) signaling pathway, fibroblast growth factor (FGF) signaling pathway and Hippo/YAP signaling pathway, etc (Fig. 1).

#### Wnt signaling pathway

The Wnt signaling pathway is a complex and multifunctional signal transduction network that involves aspects such as cell proliferation, differentiation, migration, polarity, asymmetric cell division, embryonic development, and cancer. It is an important signaling pathway that influences cell fate determination and differentiation, playing a key role in the development and regeneration of the stomach [86, 87]. The Wnt signaling pathway is typically classified into the canonical (β-catenin-dependent) and non-canonical (β-catenin-independent) pathways [88, 89]. Canonical WNT/β-catenin signaling cascades are involved in stem cell self-renewal and progenitor cell proliferation or differentiation [90–92], whereas noncanonical WNT signaling cascades are involved in the maintenance of stem cells, directed cell motility, or inhibition of canonical WNT signaling cascades [93, 94]. Almost all components of the Wnt signaling pathway are regulated by ncRNA, and different ncRNAs may regulate the Wnt signaling pathway by directly targeting major components of the Wnt signaling pathway or by targeting other mediators of non-major components, promoting or inhibiting the stemness, proliferation, invasion, metastasis, EMT, and resistance of GC cells [95]. For example, the major component of the β-catenin degradation complex, APC, can be directly regulated by miR-192, miR-215, miR-106a-3p, miR-27, miR-135b-5p, and other miRNAs to activate the Wnt signaling pathway and promote the proliferation, metastasis, and EMT of GC cells [96–99].

#### Notch signaling pathway

The Notch signaling pathway is a highly conserved intercellular signaling mechanism widely present in multicellular organisms. It regulates cell fate determination, differentiation, proliferation, apoptosis, and boundary formation through the interaction between Notch receptors and their ligands on adjacent cells. This pathway influences organogenesis and morphogenesis, maintains tissue homeostasis, and is a key route in regulating cell self-renewal, cell fate determination, and the development from embryonic to postnatal stages, as well as the homeostasis of adult cells [100, 101]. When Notch receptors bind to ligands, a series of cleavage and activation events occur, ultimately transmitting the signal to the nucleus, where it regulates the expression of downstream target genes. The Notch signaling pathway plays an important role in the zonation and differentiation of the stomach. Activation of Notch signaling in gastric epithelial cells is sufficient to induce the dedifferentiation of all lineage epithelial cells into stem cells and/or multipotent progenitor cells [102, 103]. In contrast, inhibition of the Notch pathway mainly reduces the proliferation of gastric antral stem cells and progenitor cells and increases cell differentiation, including surface mucous, deep mucous, and endocrine cells [104, 105]. Notch signaling pathway is responsible for monitoring gastric epithelial stem cell proliferation and differentiation, as well as gastric tissue growth. Polyps can form when Notch activity is uncontrolled in stem cells [104]. The Notch signaling pathway is primarily regulated by epigenetic modifications and post-translational modifications (PTMs) [106]. The intricate interplay between miRNAs and the Notch signaling pathway influences the occurrence and progression of cancer [107, 108]. For instance, miR-34a, miR-429, and miR-421 can suppress the expression of NOTCH1, thereby inhibiting the proliferation and migration of GC cells, which are malignant phenotypes [109–111]. The Notch signaling pathway is also precisely regulated by various PTMs, with ubiquitination playing a critical role. Ubiquitin ligases affect the stability and function of Notch receptors and their ligands through ubiquitination, leading to receptor endocytosis and degradation, which in turn regulates the intensity and duration of Notch signaling [112]. Other PTMs, such as phosphorylation, glycosylation, and fucosylation, also influence the activity, stability, and signal transduction of Notch receptors or ligands [113]. Collectively, these modifications ensure the precise activation and transmission of Notch signals, which are vital for determining cell fate and preventing the progression of GC.

#### Hedgehog (Hh) signaling pathway

The Hedgehog (Hh) signaling pathway plays a crucial role in animal development, controlling cell fate, proliferation, and differentiation. It is involved in the growth and differentiation of cells during embryonic development, the formation of tissues and organs, and the maintenance and homeostasis of adult stem cells. Abnormal activation of the Hh signaling pathway is closely associated with the occurrence and progression of various cancers and plays an essential role in the development of the gastrointestinal tract and the maintenance of gastric physiological functions [114]. Abnormal activation of the Hh signaling pathway promotes inflammation and carcinogenesis of gastric epithelial cells, leading to changes in the plasticity of gastric epithelial cells [115]. The main components of the Hh signaling pathway include three Hh ligands, the transmembrane receptor PTCH1, the G protein-coupled receptor SMO, and the negative regulator SUFU and transcription factors GLI1, GLI2, and GLI3 [116]. Sonic Hedgehog (Shh) signaling has been well studied in gastric physiology and pathophysiology. Gastric parietal cells secrete Shh, which contributes to epithelial regeneration during infection with Hp or at the onset of gastritis [117, 118]. The Shh signaling pathway is dysregulated, causing gastric differentiation to be disrupted, gastric acid secretion to be reduced, and GC to develop [119, 120]. In GC, abnormal Hh signaling reshapes the invasive phenotype of cancer cells, such as tumor transformation, tumor progression, metastasis, and drug resistance [121]. Due to the significant carcinogenic effect of the Hh pathway, dozens of molecular inhibitors that may inhibit the pathway have been developed in preclinical and clinical studies, mainly including SMO inhibitors, ligand-receptor inhibitors, and Gli-targeted inhibitors, etc. [122]. Different inhibitors inhibit the stem cell properties of GC cells through various mechanisms, promoting the differentiation of GC cells to a more mature state and inhibiting the invasion and metastasis of GC cells and other malignant phenotypes [115, 122].

#### TGF-β signaling pathway

The TGF-β signaling pathway is pivotal in regulating gastric tissue development, proliferation, apoptosis, differentiation, and homeostasis [123]. This pathway is categorized into two primary mechanisms: the canonical Smad-dependent pathway and the non-canonical Smad-independent pathway. TGF-β serves as a crucial inducer of EMT, with fibronectin—a marker of EMT progression—facilitating EMT through Smad 3/4-mediated TGF-β signaling [124]. TGF-β, secreted by tumor cells, is involved in promoting EMT and activating cancer-associated fibroblasts (CAFs) via paracrine signaling cascades. In a reciprocal manner, CAFs secrete additional TGF-β, further propelling EMT. The binding of extracellular TGF-β to its receptors triggers the expression of key EMT-associated genes [125, 126]. Moreover, TGF-β can stimulate non-Smad pathways, accelerating EMT progression. Concurrently, TGF-β1 activates the Wnt signaling pathway in GC cells, particularly upregulating the expression of Wnt3a, β-catenin, and CyclinD1, which fosters EMT in these cells. The TGF-β pathway has been recognized as a significant stem cell-related signaling conduit. In murine GC cells, the activation of the TGF-β pathway downregulates Sca-1 expression, a marker identified for enriching tumor stem cells. Elevated Sca-1 expression is correlated with increased resistance to cisplatin/fluorouracil chemotherapy [127]. Furthermore, TGF-β enhances the anticancer efficacy of docetaxel by promoting the differentiation of gastric CSCs.

#### FGF signaling pathway

The FGF signaling pathway plays a pivotal role in regulating the phenotypic transformation of gastric epithelial cells, influencing cell fate determination and differentiation, which are crucial for gastric development and regeneration. This pathway typically transduces signals through fibroblast growth factor receptors (FGFRs) and engages downstream signaling cascades such as RAS-MAPK/ERK and PI3K/AKT/mTOR [128]. The MAPK/ERK cascade responds to extracellular signals by activating mitogen-activated protein kinases, thereby affecting gene expression and controlling cell proliferation and differentiation. In contrast, the PI3K/AKT/mTOR cascade promotes cell survival through the activation of AKT kinase and subsequently influences cell cycle, protein synthesis, and cellular metabolism via mTOR [129]. The FGF signaling pathway is subject to intricate regulation, with various signaling molecules potentially modulating FGF signaling either by directly targeting key components of the pathway or by targeting other mediators that are not primarily associated with it. This modulation can promote or inhibit the stemness, proliferation, invasion, metastasis, EMT, and drug resistance of GC cells [130]. For instance, the activation of FGFR can be directly regulated by microRNAs such as miR-125b and miR-200c, which activate the FGF signaling pathway and promote the proliferation, migration, and EMT of GC cells [131].

#### Hippo/YAP signaling pathway

The Hippo/YAP signaling pathway is a highly conserved signaling pathway in evolution, controlling organ size by regulating cell proliferation, apoptosis, and stem cell self-renewal, and its dysregulation is closely related to cancer development [132]. It plays a key role in regulating the phenotypic transformation and differentiation levels of gastric epithelial cells by modulating the activity of its main effectors YAP1/2 and TAZ, affecting cell fate determination and differentiation. In gastric adenocarcinoma, the expression of YAP is significantly increased, with higher cytoplasmic YAP levels in the early tumor stage and higher nuclear YAP levels in the advanced tumor stage. The accumulation of nuclear YAP is correlated with lower survival rates, especially in early GC patients [133]. YAP mediates the peritoneal carcinomatosis in advanced gastric adenocarcinoma patients. The oncogenic protein Myc has been identified as a key downstream target of YAP in initiating gastric tumorigenesis. The upregulation of YAP is significantly associated with HP infection status and the progression of GC, including tumor size and staging [134]. Conversely, in 61% of GC samples, the transcriptional level of VGLL4, a negative regulator of the Hippo signaling pathway, is downregulated, showing a negative correlation with GC progression, including tumor size, staging, and lymph node metastasis [135]. The YAP/VGLL4 ratio may serve as a potential biomarker for GC. In a study of 86 advanced GC patients receiving first-line chemotherapy, a significant correlation between nuclear TAZ expression and Wnt mutation was discovered. Patients with tumors exhibiting combined nuclear TAZ expression and Wnt mutations have a significantly increased risk of disease progression and lower overall survival. Additionally, YAP/TAZ inhibitors can block the EMT process in GC cells, inhibiting tumor cell invasion and metastasis [136].

## The therapeutic potential of cellular plasticity in the induced differentiation of gastric cancer

Traditional cancer treatments aim to kill rapidly proliferating tumor cells and can assess the treatment’s effectiveness by directly observing changes in tumor volume over a relatively short period of time. However, the evaluation of differentiation therapy effectiveness does not involve changes in cell number or tumor total volume. The dedifferentiation of GC cells is related to the loss of gastric epithelial cell function and invasive morphology, which can be confirmed by detecting gastric cell differentiation markers such as functional proteins and surface markers. Therefore, detecting gastric epithelial cell differentiation markers and the morphological transformation of tumor cells into normal cells can help determine the successful induction of differentiation in GC cells. GC can benefit from differentiation therapy in several ways, including the reversal of malignant phenotypes, restoration of normal epithelial function, enhancement of immune function, and increased resistance to conventional cancer treatments. Therefore, it provides a new strategy for the clinical treatment of GC and is expected to become a radical cure for GC.

**Fig. 2:**
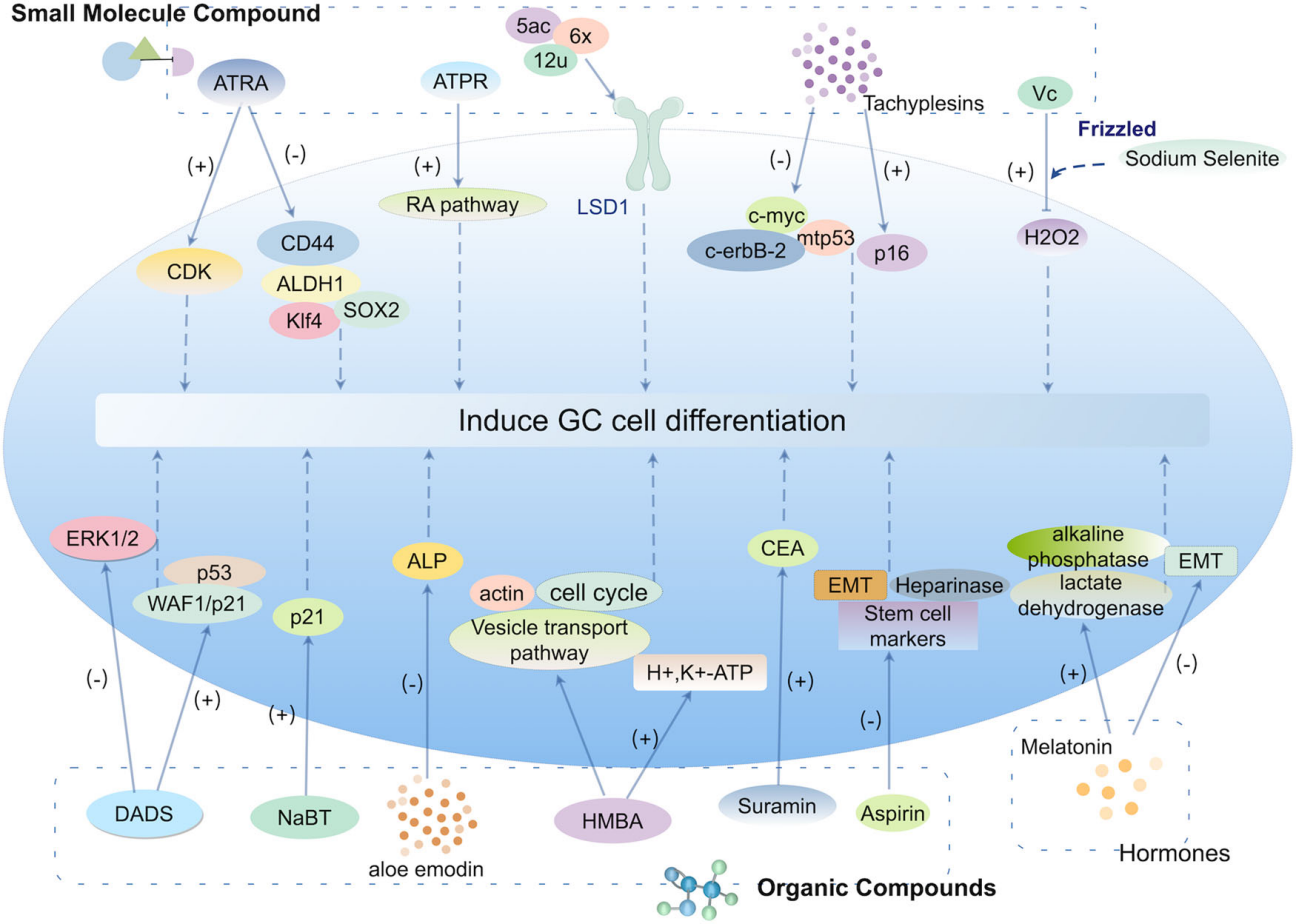
The therapeutic potential of cellular plasticity in the induced differentiation of gastric cancer. Small molecule compounds, organic compounds, and hormones have been applied to induce targeted differentiation of gastric cancer cells (by Figdraw).

Studies have shown that despite the presence of extensive genomic alterations in cancer cells, phenotypic reversal can still be achieved [137]. The plasticity of tumor cells provides a fundamental premise for remodeling and reshaping tumor cells. In recent years, therapeutic strategies aimed at inducing the re-differentiation of tumor cells have become a hot topic in cancer research [4, 138]. The strategies for differentiation therapy currently under investigation demonstrate multifaceted potential, including small molecule compounds, organic compounds, and hormones. The main features of these strategies include: (1) inducing differentiation of tumor cells and inhibiting their malignant biological properties; (2) reversing progenitor cell characteristics, inhibiting tumor growth and metastasis; (3) inhibits the potential of GC cell stem cell properties and restores part of gastric function. For instance, melatonin, a hormone, has been shown to increase the expression of alkaline phosphatase and lactate dehydrogenase, significantly promoting the differentiation of GC cells, and also exhibits a significant inhibitory effect on the epithelial-mesenchymal transition (EMT) process. This suggests that hormones may also play an important role in the differentiation therapy of GC cells. To date, mature therapies that use cell plasticity mechanisms to inhibit or reverse the progression of GC in patients have not been resoundingly developed. This article reviews the current research status of targeted induction of GC cell differentiation therapy strategies (Fig. 2, Table 3) and provides theoretical support for the development of highly specific GC differentiation therapy in the future.

**Table 3:** Drugs targeted to induce differentiation of gastric cancer cells.

Classification	Target	Function	Mechanism	Reference
Small molecule compounds				
	ATRA	ATRA induces the differentiation of tumor-initiating cells in GC, inhibits the formation and growth of tumor spheres, and induces the differentiation of tumor spheres.	ATRA arrests the cell cycle by up-regulating cyclin-dependent kinase inhibitors (CDKs), down-regulates cell cycle progression activators, down-regulates the expression of tumor stem cell markers CD44 and ALDH, and stem cell genes such as Klf4 and Sox2.	[139]
	ATPR	Inducing cell differentiation and apoptosis.	ATPR depends on the retinoic acid signaling pathway to regulate the expression of genes related to cell differentiation and apoptosis.	[140, 141]
	LSD1 inhibitors	Induce apoptosis and differentiation of GC cell line MGC-803 cells, inhibit migration, and induce cell stemness and enhance the immune response.	Inhibit LSD1 expression in tumors to inhibit cancer progression.	[142–146]
	Tachyplesins	Inhibits gastric cancer cell growth and mitosis.	Down-regulated the expression levels of cancer genes c-erbB-2, c-myc, and mtp53 proteins, while up-regulating the expression level of anti-cancer gene p16 protein.	[147]
	Vitamin C	The malignant characteristics of cells were significantly reduced, the colony formation potential of cell differentiation indicators was reduced, and human cancer cells were induced to redifferentiate and cell growth was inhibited.	It can enhance the activity of antioxidant enzymes, induce the formation of H2O2, and change the cellular redox state.	[148, 149]
Organic Compounds				
	Metformin	Induce GC cell differentiation, resulting in slow tumor growth, and reduced the self-renewal capacity of GC stem cells.	Induced cell cycle arrest, inhibited cell proliferation, and reduced the number of tumor spheres.	[150–152]
	DADS	DADS can inhibit the growth of MGC803 cells and induce the differentiation and apoptosis of gastric cancer BGC-823 cells.	DADS reduced the phosphorylation of ERK1/2 protein and inhibited the activation of ERK1/2 in a concentration-dependent manner, The p21 and p53 genes may play an important role in the differentiation of gastric cancer cells induced by DADS.	[160, 161]
	NaBT	Reverse the malignant phenotype of AGS, cell proliferation was significantly inhibited, and the structure of AGS cells changed greatly.	NaBT can induce G1 phase arrest of AGS cells by up-regulating the expression of p21 and inhibiting cell proliferation.	[153, 154]
	Aloe emodin	Aloe emodin inhibits the growth of cancer cells in a dose-dependent manner, and reduces the activity of alkaline phosphatase (ALP).	Through cell cycle interruption and induction of differentiation.	[155]
	HMBA	Induces morphological differentiation and cell cycle arrest in human GC BGC-823 cells, inducing differentiation of GC cells.	May involve complex processes, including signal networks of connecting vesicle transport, cell skeleton remodeling other than morphological differentiation, and cell cycle G1 arrest, induces G0/G1 arrest and up-regulation of proton pump.	[156–158]
	Suramin	Inhibits the growth of SNU-16 cells and induces the differentiation of SNU-16 cells.	Induces the development of intracytoplasmic lumen and intercellular lumen, forms a large number of microvilli and frequent desmosomes, releases CEA, and inhibits cell ectopic transplantation.	[162]
	Aspirin	Induces differentiation of GC cells into non-proliferating cells, Inhibits cell proliferation.	Decreased the mRNA expression of EMT-related molecules, stem cell markers and heparinase, and induced cell cycle arrest in the G0/G1 phase of KATO-III cells.	[159]
Hormones				
	Melatonin	Promote the differentiation of GC cells.	Increase the expression of alkaline phosphatase and lactate dehydrogenase, inhibits EMT.	[163, 164]

### Small molecule compounds

In the ongoing quest for novel therapeutic strategies against GC, researchers are exploring a diverse array of compounds and mechanisms that target various aspects of cancer cell behavior, such as the induced differentiation properties of retinoic acid derivatives, the epigenetic regulation of differentiation by LSD1 inhibition, the antimicrobial potential of tachyzoin, and the redifferentiation role of vitamin c. Each section delves into the specific mechanisms by which these agents may suppress tumor growth, induce cell differentiation, and combat the stemness and drug resistance often associated with GC.

All-trans retinoic acid (ATRA) is a widely used differentiation inducer in clinical practice. Currently, ATRA has become the first-line drug for the treatment of acute promyelocytic leukemia and is widely used in the prevention and treatment of solid tumors such as breast cancer, thyroid cancer, non-small cell lung cancer, prostate cancer, and cervical cancer. However, the therapeutic effect of retinoic acid compounds on GC is still in the research stage. Studies have found that ATRA induces the differentiation of tumor-initiating cells in GC, inhibits the formation and growth of tumor spheres [139]. ATPR, a derivative of ATRA, has further enhanced the therapeutic effect of GC [140]. ATPR depends on the retinoic acid signaling pathway to regulate the expression of genes related to cell differentiation and apoptosis, inducing cell differentiation and apoptosis. When GC cells have poor differentiation, ATPR is more likely to induce cell differentiation, and when GC cells have good differentiation, ATPR is more likely to induce cell apoptosis. ATPR has a better anti-tumor effect on GC cells than ATRA and has minimal inhibitory effects on normal cells. ATPR has a better ability to induce differentiation in GC cells with poor differentiation at concentrations of 3.0 to 6.2 μM [141]. The results suggest that novel retinoic acid drugs have the potential to be effective therapeutic agents for GC differentiation induction therapy.

Lysine-specific histone demethylase 1 (LSD1) is an enzyme that removes methylation modifications on histone H3K4 and H3K9. There is a significant upregulation of LSD1 in GC tissues and an association between this gene and stemness and drug resistance in the GC [142, 143]. LSD1 is currently considered an important epigenetic target for cancer therapy [144], and a series of multi-target preparations and compounds that inhibit LSD1 expression in tumors to inhibit cancer progression have been developed, several of which have entered clinical trials [145]. In GC patients, the therapeutic effect of LSD1 inhibitors has also been recognized. For example, Qi-Sheng Ma et al. developed an LSD1 inhibitor based on trans-cyclopropylamine derivative 12 u, which can induce apoptosis and differentiation of GC cell line MGC-803 cells, inhibit migration, and induce cell stemness [144]. A series of anthraquinone LSD1 inhibitors, such as 5ac and 6x, have also shown significant inhibitory activity against LSD1 and can inhibit the stemness and migration of GC cells and enhance the immune response [146].

Tachyplesins are a family of peptide small molecules found in granulocytes in horseshoe crab blood. Treatment of GC cell line BGC-823 with tachyplesins down-regulated the expression levels of cancer genes c-erbB-2, c-myc, and mtp53 proteins, while up-regulating the expression level of anti-cancer gene p16 protein. The growth inhibition rate of cells was 62.29%, and the maximum mitotic index decreased by 51.95%. Tachyplesins can can be used as a differentiation inducer of GC cells [147].

Vitamin C (VC), also known as ascorbic acid or L-ascorbic acid, is a water-soluble vitamin, with the molecular formula C6H8O6. After treating GC cells with VC, the malignant characteristics of the cells were significantly reduced, and the potential of the colony-forming unit in the cell differentiation index was reduced by up to 90.2%. Further mechanistic studies showed that VC induces growth inhibition and redifferentiation of GC cells by producing hydrogen peroxide [148]. When combined with sodium selenite, the effects are more pronounced, enhancing the activity of antioxidant enzymes, inducing H_2_O_2_ formation, and altering the cellular redox state. This combination ultimately promotes cancer cell redifferentiation and inhibits cell growth. The combined application of VC and sodium selenite may be an effective anticancer agent for the treatment of human cancer [149].

### Organic compounds

Organic compounds such as metformin, sodium butyrate (NaBT), aloe emodin, hexamethylene bisacetamide (HMBA), aspirin, diallyl disulfide (DADS), and suramin can modulate cellular processes, including the cell cycle and differentiation. This section highlights a range of compounds, from common medications like Metformin and Aspirin to natural products and specialized chemicals, that have shown promise in inducing cell cycle arrest and differentiation in GC cells. Each compound presents a unique mechanism of action that could potentially be harnessed to slow tumor growth, reduce the self-renewal capacity of cancer stem cells, and transform the malignant phenotype of GC cells.

#### Compounds inducing cell cycle arrest and differentiation

Metformin is the first-line drug for type 2 diabetes. Recently, it has been found that metformin can significantly inhibit tumor growth when administered in a fasted state [150]. Metformin induced cell cycle arrest, inhibited cell proliferation, and reduced the number of tumor spheres, revealing its ability to target the stemness of GC cells [151]. Further exploration of the expression of differentiation markers (Kruppel 4 and MUC5AC) confirmed this effect, and metformin was able to induce GC cell differentiation, resulting in slow tumor growth, and reduced the self-renewal capacity of GC stem cells [152].

Sodium butyrate (NaBT) is a differentiating inducer for GC cells. After treating GC cell BGC-823, cell growth was significantly inhibited, the proportion of G(0)/G(1) phase cells increased, and the proportion of S-phase cells decreased, with a significant decrease in the mitotic index [147]. After treating GC cell AGS with different concentrations of NaBT, cell proliferation was significantly inhibited, and the structure of AGS cells changed greatly. NaBT can induce G1 phase arrest of AGS cells by up-regulating the expression of p21 and inhibiting cell proliferation. This can, to a certain extent, reverse the malignant phenotype of AGS cells [153, 154].

Aloe emodin is a novel active compound discovered in the root and rhizome of rhubarb. After treating GC cell MGC-803 with aloe emodin, it was found that aloe emodin inhibits the growth of cancer cells in a dose-dependent manner, increases the proportion of cells in the S-phase and high ploidy level (>G2/M) in the cell cycle, and reduces the activity of alkaline phosphatase (ALP), which is a cell differentiation index. These results suggest that aloe emodin has potential therapeutic value for GC, and its mechanism is through cell cycle interruption and induction of differentiation [155].

Hexamethylene bisacetamide (HMBA) induces morphological differentiation and cell cycle arrest in human GC BGC-823 cells, inducing differentiation of GC cells. The molecular mechanism by which BGC-823 cells respond to HMBA-induced differentiation may involve complex processes, including signal networks of connecting vesicle transport, cell skeleton remodeling other than morphological differentiation, and cell cycle G1 arrest [156]. In addition, treatment of GC cells with HMBA induces G0/G1 arrest and up-regulation of proton pump, a marker of cancer differentiation [157]. Before and after HMBA treatment, nuclear matrix proteins undergo marked changes in expression and intracellular distribution, and nuclear matrix proteins influence cancer cell differentiation [158]. HMBA, as a GC cell differentiation inducer, has broad application prospects.

Aspirin, also known as acetylsalicylic acid, is an organic compound with the chemical formula C_9_H_8_O_4_. After treating GC cell KATO-III with aspirin, a decrease in the expression of EMT-related molecules, stem cell markers, and acetylated HDACs mRNA was observed. Aspirin also has an inhibitory effect on cell proliferation. Moreover, aspirin induces G0/G1 phase cell cycle arrest in KATO-III cells. In summary, aspirin induces the differentiation of GC cells into non-proliferating cells and provides possibilities for new drug design [159].

#### Compounds inhibiting cell growth and inducing differentiation

DDADS is the main component of garlic. GC cell MGC803 treated with DADS shows a lower nuclear-to-cytoplasmic ratio and tends to form glandular and intercellular junctional structures. This suggests that DADS induces the differentiation of MGC803 cells [160, 161].

Suramin is a reversible, competitive protein tyrosine phosphatase (PTPases) inhibitor. The development of intracellular and intercellular cavitational structures, abundant microvilli, and frequent bridge grains were observed in GC cell SNU-16 cultures containing suramin. In the presence of suramin, the amount of CEA released by the cells increased. Suramin inhibits ectopic transplantation, and transplantation from cultures containing suramin showed a much higher degree of differentiation than those without suramin. Studies have shown that suramin inhibits the growth of SNU-16 cells and induces the differentiation of SNU-16 cells [162].

### Hormones

Hormones play important physiological roles in human body, mainly regulating growth and development, nutrient metabolism, affecting nervous system, reproductive system, cardiovascular and kidney functions,. Recent studies have found that certain hormones are involved in the remodeling process of gastric cancer cells. For example,

melatonin administration can increase the expression of alkaline phosphatase and lactate dehydrogenase and significantly promote the differentiation of GC cells [163]. Using a gastric adenocarcinoma cell line, it was found that melatonin inhibits significantly EMT process [164]. At present, the studies on hormone involvement in induced differentiation of gastric cancer are very limited, and further exploration would be necessary.

## Conclusion and prospects

Cellular plasticity refers to the determination, transformation, and reshaping of cell fate. Gastric epithelial cells have obvious plasticity, and they can change their differentiation state through forms such as transdifferentiation, dedifferentiation, and blocked differentiation under different physiological and pathological conditions, showing different differentiation phenotypic characteristics, including normal gastric mucosal epithelium, SPEM, IM, EMT, and GC. The activation of transcription factors, abnormal epigenetic regulation, infection, acidic environment, and the combined effect of signaling pathways such as Wnt, Notch, Hedgehog, etc., constitute a complex network that directly or indirectly regulates the plasticity of gastric epithelial cells. The plasticity of cancer stem cells is closely related to the occurrence, development, invasion, and metastasis of GC. Currently, GC treatment strategies based on the mechanism of cellular plasticity have become a research hot spot, including the development of small molecules, organic compounds, hormones, etc. As a double-edged sword, plasticity is beneficial for the repair and regeneration of gastric epithelium, as well as the development and transformation of GC. By understanding the connotation of gastric epithelial cell plasticity, the mechanism of adaptive changes in gastric epithelium under different environments and stimuli can be revealed. By focusing on the crucial factors and processes that affect gastric epithelial plasticity, we can promote the differentiation of GC cells, prevent the occurrence and progression of GC, boost sensitivity and immune response of GC, and decrease recurrence and drug resistance. However, research related to the plasticity of gastric epithelial cells still faces some challenges and problems, which need to be addressed and improved in future research. First, there are still gaps in our understanding of cellular dynamics, particularly a lack of knowledge about the dynamic processes of cell state transitions at the single-cell level. Both epithelial and mesenchymal states cannot be clearly linked to stem cell states [165]. Although single-cell RNA sequencing technology can provide a detailed view of cellular heterogeneity, there are still technical limitations in single-cell and multi-omics analyses, such as the potential loss of cell types during cell preparation [131], These technical limitations may affect the accurate identification and characterization of cell subpopulations. Moreover, current in vitro and in vivo models are insufficient in simulating the dynamic changes and spatiotemporal distribution of gastric epithelial cell plasticity. Although advancements in spatial transcriptomics technology will allow future research to determine how changes in cellular phenotypes during disease progression affect the interaction between the stroma and epithelial environment, the application of this technology in gastric cancer research is still in its infancy. The clinical value and application of gastric epithelial cell plasticity require more clinical trials and evaluations for validation. In summary, in future research, an in-depth exploration of the plasticity of gastric epithelial cells will provide a more solid foundation for us to solve the remaining problems and promote clinical applications. This review on the plasticity of gastric epithelial cells and the induction of GC cell differentiation provides valuable theoretical references and profound research insights for the basic research and clinical applications of GC cell differentiation reprogramming and precision treatment fields.
